# The impact of sensor application site on continuous noninvasive haemoglobin trends

**DOI:** 10.1016/j.csbj.2025.07.013

**Published:** 2025-07-12

**Authors:** Audrius Andrijauskas, Povilas Andrijauskas, Darius Dilijonas, Ieva Jovaisiene, Arūnas Valaika, Axel Kerroum, Karolis Urbonas, Nadezda Scupakova, Lina Puodžiukaite, Vaidotas Marozas, Edgaras Stankevicius, Mindaugas Budra, Gintaras Kalinauskas, Darius Cincikas, Edvin Vasilevski, Tomas Jovaisa

**Affiliations:** a Clinic of Anaesthesiology and Intensive Care, Institute of Clinical Medicine, Faculty of Medicine, Vilnius University, M.K. Čiurlionio str. 21, Vilnius LT-03101, Lithuania; b II Department of Anaesthesiology and Intensive Care, Vilnius University Hospital Santaros Klinikos, Vilnius, Lithuania; c Institute of Social Sciences and Applied Informatics, Kaunas Faculty, Vilnius University, Muitinės str. 8, Kaunas LT-44280, Lithuania; d Clinic of Cardiac and Vascular Diseases, Institute of Clinical Medicine, Faculty of Medicine, Vilnius University, M.K. Čiurlionio str. 21, Vilnius LT-03101, Lithuania; e Department of Anesthesiology, Lausanne University Hospital (CHUV), Rue du Bugnon 46, Lausanne 1005, Switzerland; f Faculty of Electrical and Electronics Engineering, Kaunas University of Technology, Studentu str. 50, Kaunas LT-51368, Lithuania; g Institute of Physiology and Pharmacology, Lithuanian University of Health Sciences, Kaunas 44307, Lithuania; h Faculty of Medicine, Vilnius University, M.K. Čiurlionio str. 21, Vilnius LT-03101, Lithuania; i Biomedical Engineering Institute, Kaunas University of Technology, K. Barsausko str. 59, Kaunas LT-51423, Lithuania

**Keywords:** Noninvasive haemoglobin, Cardiac surgery, Monitoring, PPG, Transcapillary fluid shift

## Abstract

**Purpose:**

This prospective observational clinical study investigated discrepancy between noninvasive continuous haemoglobin (SpHb, Masimo Corp., Irvine, CA) measurements with sensors placed on three fingertips during elective cardiac surgery.

**Materials and methods:**

The study included 12 adult patients who underwent elective coronary artery bypass grafting (CABG) under cardiopulmonary bypass (CPB). Three identical versions of SpHb sensors were placed on the fingers of the same hand and connected to dedicated Masimo Radical-7 monitors. Data were continuously recorded throughout the surgery. Multiple group comparison tests—Kruskal—Wallis and Alexander—Govern tests—were used to compare three sets of SpHb data (trends). Bland—Altman and Giavarina plots were used to compare the SpHb data with invasive arterial haemoglobin (Lab-Hb) data obtained at clinically relevant time points chosen at the discretion of the cardiac anaesthesiologist, who was blinded to the study protocol.

**Results:**

There was a good correlation between the time-matched SpHb data from the three sensors at 253 time points (r = 0.86–0.95), but there was a significant difference between the SpHb values obtained from each sensor and the average SpHb value of all 3 sensors (p < 0.05) at each time point. The absolute difference between the SpHb value obtained from each sensor and the average SpHb value was 0.69 g/dl g/dl (SD 1.49 g/dl). The accuracy and precision of the average SpHb for estimating Lab-Hb was higher compared to the SpHb values obtained from two out of three individual sensors (sn1 and sn3) and very similar compared to values obtained from sensor sn2.

**Conclusion:**

The sensor placement site can impact the SpHb measurement causing discrepancy between SpHb measured in different fingers. The average SpHb from three fingertips appears to be more accurate and precise in estimating Lab-Hb. However, more advanced sensors are needed.

## Graphical Abstract

**Figure:**
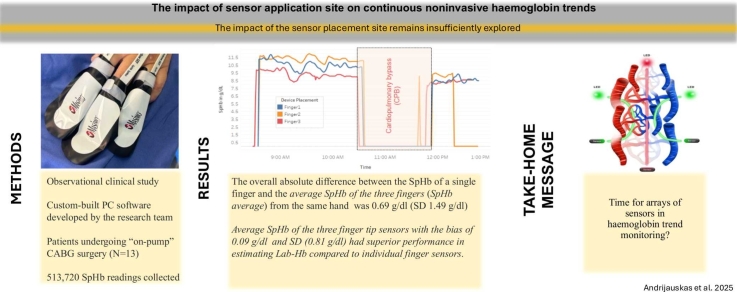

## 1 Introduction

Haemoglobin measurement is crucial for screening and treatment across various medical conditions. Invasive techniques are limited by discomfort, delayed results, and infection risk. While various noninvasive methods and devices have been developed, only one commercially available technique - the pulse CO-Oximetry used by the Masimo Radical-7 monitor (Masimo Corp., USA) [1] - provides continuous measurements of total haemoglobin (SpHb). A prototype of conceptually similar haemoglobin monitor developed by the University of Limerick (Ireland) [2] has not been commercialized.

Continuous haemoglobin monitoring is particularly important for estimating perioperative blood loss in cardiac surgery, however, the accuracy and especially precision of noninvasive haemoglobin (SpHb) monitoring using Radical-7 monitor in estimating invasive haemoglobin concentration (Lab-Hb) remain inadequate and represent a persistent limitation [3], [4].

Scientific investigations summarizing the accuracy and precision of point-of-care testing and monitoring devices in estimating Lab-Hb were presented in a 2019 review; the results from 18 invasive and 5 noninvasive devices revealed a gap between invasive and noninvasive techniques, with no discernible improvement observed between 2010 and 2018 [5]. Since the technology appears to be internally flawed rather than fundamentally limited [1], overcoming common obstacles may be sufficient to achieve clinically trustable noninvasive haemoglobin monitoring.

Since 2009, several authors of the present article have dedicated their time to exploring ways to improve the accuracy and precision of SpHb monitoring when Radical 7 became commercially available. Trials in healthy volunteers and patients undergoing major orthopaedic surgery [6], [7], [8], [9] have revealed that changes in transcapillary fluid equilibration are very likely to impact the accuracy of SpHb in estimating invasive venous and arterial haemoglobin concentration. An anatomy and physiology-based transcapillary reflux model [8] was proposed to explain that theory, as well as to explore its feasibility for the use of SpHb in monitoring fluid accumulation and detecting imminent oedema. Improvement of perioperative accuracy and precision of SpHb measurements requires further studies, particularly in cardiac surgery settings [3], [4], [10], [11].

One factor that remains underexplored is the impact of SpHb sensor placement site, as the Radical-7 monitor has not been evaluated using sensors on multiple fingertips simultaneously. The present work, as a continuation of the above-mentioned research, explores the impact of SpHb sensor placement site as theoretically possible source of error in estimates.

Capillary density and their perfusion are different even in adjacent segments of the same tissue, e.g. derma. Thus, since perfusion and local metabolism related transcapillary fluid shifts are affecting haemoglobin concentration, we hypothesize that SpHb measured in different fingertips is inherently different.

To address this hypothesis, the present prospective observational clinical study investigated the impact of sensor application site on SpHb trends by exploring discrepancy between measurements by sensors placed on three adjacent fingers during coronary artery bypass grafting (CABG) surgery under cardiopulmonary bypass (CPB). Additionally, this study explored whether averaging SpHb values from multiple fingers could provide a more accurate and precise estimate of haemoglobin concentration.

## 2 Materials and methods

This trial was approved by the Vilnius regional biomedical research ethics committee (protocol number 2024/4–1579–1038) and conducted in accordance with the Declaration of Helsinki. Patient confidentiality was maintained through deidentification and secure data storage.

### 2.1 Research objectives and hypotheses

The primary endpoint of the study was to determine the impact of sensor placement site on discrepancies in SpHb measurements by evaluating the correlation and differences between simultaneous SpHb estimates obtained from three fingers of each patient; both individual and pooled data from 12 subjects were used for analysis. The secondary endpoint was to assess the accuracy and precision of SpHb in estimating laboratory haemoglobin (Lab-Hb) by comparing SpHb to Lab-Hb values. For this analysis, both the average SpHb value (AvgSpHb) from the three fingers and the SpHb values from individual fingers of the same subject were considered.

### 2.2 Study design

The sample size of 12 subjects was selected based on recommendations for pilot observational clinical trials because *ad hoc* estimates could not be performed since assessment of SpHb accuracy and precision in several fingers in clinical settings was not reported at the time. The study did not involve interventions beyond the institution’s standard of care, and participation in the study did not affect surgical or postoperative management.

### 2.3 Eligibility and enrolment

Consecutive adult patients scheduled for elective CABG surgery with CPB at Vilnius University Hospital Santaros Klinikos were invited to participate in the trial. The inclusion criterion was patients aged ≥ 18 years who signed an informed consent form. The exclusion criterion was patients who refused to participate. Patients were provided with detailed written information about the trial, including the purpose, procedures, risks, and potential benefits. All participants provided written informed consent.

### 2.4 Methods and technologies

Custom-built PC software was developed by the research team using Python and installed in the study-dedicated PC for recording and *post hoc* analysis of relevant data. Three Radical-7 monitors were integrated via the serial communication standards RS-232C and USB. The suffixes _sn1, _sn2, and _sn3 denote the Radical-7 devices used for measurements.

### 2.5 Monitoring setup and data recording

**Fig. 1:**
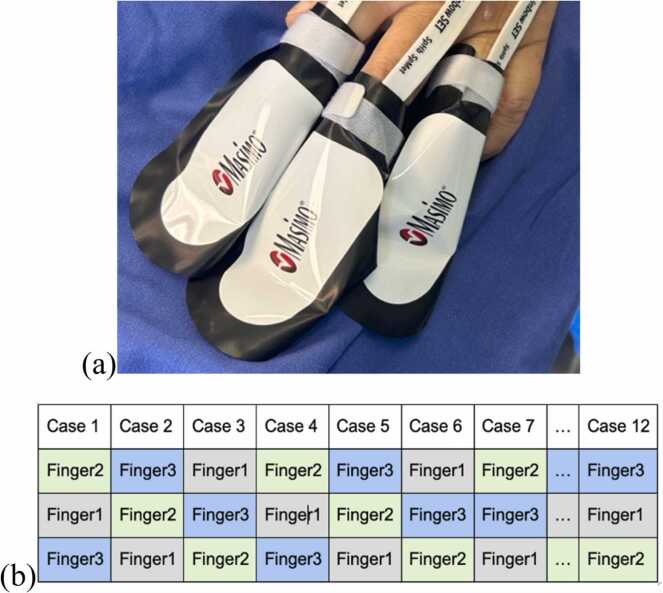
SpHb sensor application. (a) Three SpHb sensors were applied to the fingernails of the second, third, and fourth fingers of the same hand, and standard protective sleeves provided by the manufacturer were used to prevent light interference. (b) A predefined selection of combinations in which the monitor is connected to a specific finger was used to ensure a variety of possible combinations per device and fingers.

**Fig. 2:**
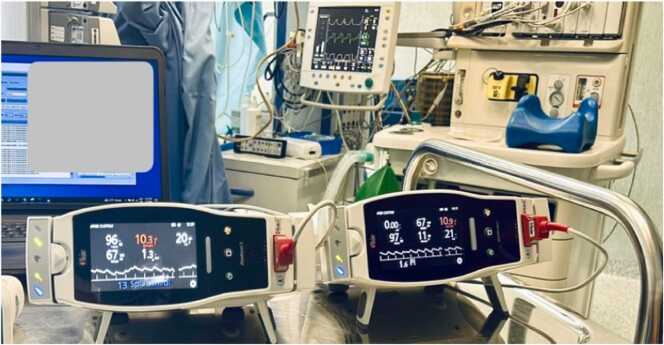
Three Radical 7 monitors (Masimo Corp., USA) were connected to three SpHb sensors on the hand of the same subject.

Upon arrival to the theatre, standard vital sign monitoring commenced (ECG, pulse oximetry, regional brain oximetry baseline) for each patient. Before the induction of anaesthesia and the start of intravenous fluid infusion, three SpHb sensors RD rainbow SET™-2 Adt (Masimo Corporation, USA) were placed on the fingertips of the second (index), third (middle), and fourth fingers of the same hand (Fig. 1a). With the aim of preventing artefacts due to interference from light from nearby sensors, the standard protective sleeves provided by the manufacturer were applied to each finger (Fig. 1a). Each sensor was connected to a separate Radical 7 monitor, and calibration was performed (Fig. 2).

**Fig. 3:**
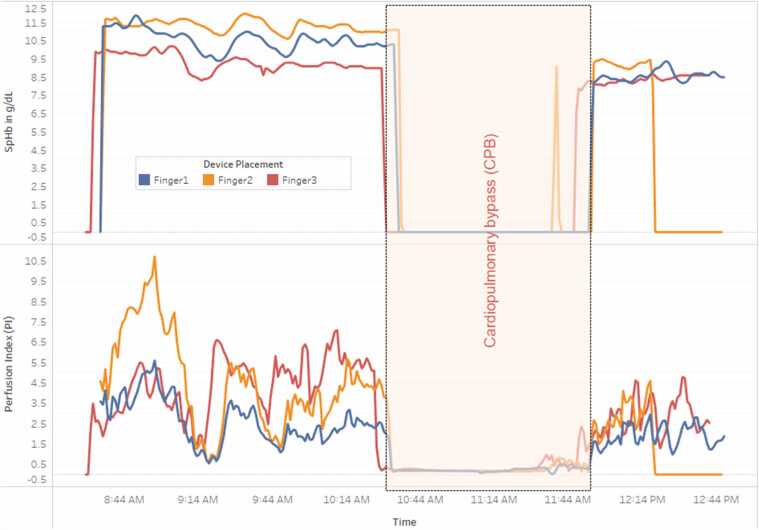
Illustration of a single case showing continuous intraoperative SpHb and PI readings from three different fingers (Finger 1 — blue lines; Finger 2 — orange lines; Finger 3 — red lines) recorded simultaneously throughout the entire surgery. X-axis: time; Y-axis: g/dL for SpHb and numerical values for PI. The cardiopulmonary bypass (CPB) period (non-pulsatile period) is marked with a light red background.

A predefined selection of combinations in which the monitor is connected to a specific finger was used to ensure a variety of possible combinations per device and fingers (Fig. 1b). Radical 7 monitors were connected to the PC, and the SpHb data were recorded. The data were recorded in real time every second via dedicated PC software. The period during CPB is shown in Fig. 3. The time points at which the arterial blood samples were drawn for ABG analysis (RAPID Point 500 Blood Gas Analyser, Siemens, Germany) to determine Lab-Hb were recorded for post hoc evaluation of SpHb accuracy and precision.

The study included three observation phases: pre-CPB, on-CPB, and post-CPB (see Supplemental Material (Appendix A) for detailed protocols).

### 2.6 Data management and analysis

The data, including demographic details and SpHb and Lab-Hb data, were securely stored in an electronic MySQL 11.3 database. The analysis was conducted using Python 3.12.4, Tableau 2024.2.2, and SQL.

The post hoc data analysis followed these steps:

1. The data were cleaned and transformed using Structured Query Language (SQL), a standard language for database access and manipulation [12] (see Supplemental material (Appendix B)).
2. Tableau, a data visualization tool, was used to explore the data (Fig. 3).
3. Python, a high-level general-purpose programming language [13], was employed for statistical analysis and hypothesis testing (see Supplemental material (Appendix B)).
4. Bland—Altman (B&A) analysis was used to assess the agreement between SpHb (averaged and individual sensor values) and Lab-Hb (see Supplemental material (Appendix B)). This determined bias (mean difference) and limits of agreement (LOA; bias ± 1.96 SD of differences). Outliers, defined as SpHb values outside the manufacturer's declared accuracy range (± 1.0 g/dl), were also calculated.
5. Giavarina analysis was performed (see Supplemental material (Appendix B)). This analysis is identical to B&A analysis except that it accounts for heteroscedasticity. It does this by using percentage differences (relative to the means) on the y-axis instead of raw differences [14].
6. Confidence Intervals (CI) statistical analysis was performed to check how number of samples of Lab-Hb would affect the confidence of secondary experiment result (the agreement between SpHb (averaged and individual sensor values) and Lab-Hb) (see Supplemental material (Appendix B)).

## 3 Results

Thirteen patients were enrolled from June to August 2024. In one patient, the Radical 7 monitor could not provide SpHb data because of unknown issues. Thus, 12 subjects completed the study. The SpHb values were recorded every second, resulting in a total of 513,720 values; the median data recording time was 253 min, with an interquartile range (IQR) of 233–266. 22 Lab-Hb values were recorded and time-stamped (during Pre-CPB and Post-CPB periods). Patient characteristics and recorded data are presented in Table 1.

**Table 1:** Patient data.

	Women n = 4	Men n = 8	Total n = 12
Age; y	67(6)	61(7)	63(7)
ASA physical status			
3	4 (100 %)	8 (100 %)	
Lab-Hb; g/dl			
All during operation (n = 45)	8.7 (8.2–11.4)	11.5 (9.7–12.4)	11.2 (9.3–12.2)
Pre-CBP or post-CPB (n = 22)	11.4 (11.0–13.2)	12.2 (11.2–13.4)	11.8 (11.0–13.4)
SpHb *vs.* Lab-Hb comparison; g/dl (n = 22);			
SpHb Device 1 (sphb_sn1)	11.2 (10.6–11.9)	11.1 (10.7–11.6)	11.2 (10.7–11.6)
SpHb Device 2 (sphb_sn2)	10.6 (9.3–11.7)	11.4 (11.2–12.5)	11.4 (11.1–11.9)
SpHb Device 3 (sphb_sn3)	10.7 (9.0–11.4)	11.3 (10.8–13.0)	11.3 (10.3–12.2)
SpHb Avg (sphb_avg_clean)	10.8 (9.3–11.4)	11.1 (10.9–12.4)	11.1 (10.8–12.0)
Radical Device Measurements			
SpO2	98.0 (97.0–100.0)	98.0 (92.0–99.0)	98.0 (95.0–99.0)
Heart rate; bpm	72.0 (61.0–94.0)	60.0 (44.0–72.0)	64.0 (51.0–77.0)
Perfusion index; PI	1.7 (0.3–3.5)	1.1 (0.1–3.0)	1.2 (0.2–3.1)
SpHb	9.2 (0.0–10.8)	10.3 (0.0–11.8)	9.8 (0.0–11.5)
Oxygen Content; SpOC	0.0 (0.0–13.0)	0.0 (0.0–15.0)	0.0 (0.0–14.0)
Pleth Variability Index; PVI	10.0 (0.0–19.0)	12.0 (0.0–20.0)	11.0 (0.0–20.0)
Sensor localization and number of measurements	Finger 1: 115800 (72.3 %) Finger 2: 29160 (18.2 %) Finger 3: 15240 (9.5 %)	Finger 1: 148620 (42.0 %) Finger 2: 102660 (29.0 %) Finger 3: 102240 (28.9 %)	Finger 1: 264420 (51.5 %) Finger 2: 131820 (25.7 %) Finger 3: 117480 (22.9 %)
Measurement minutes (points for statistical analysis)	250 (236, 263)	253 (233, 269)	253 (233, 266)
Pre-CPB and Post-CPB values			
SpO2	150975 (94.2 %)	274513 (77.7 %)	425488 (82.8 %)
Heart rate; bpm	150428 (93.9 %)	271196 (76.7 %)	421624 (82.1 %)
Perfusion index; PI	150952 (94.2 %)	274352 (77.6 %)	425304 (82.8 %)
SpHb	108222 (67.6 %)	222949 (63.1 %)	331171 (64.5 %)
Oxygen Content; SpOC	77230 (48.2 %)	149963 (42.4 %)	227193 (44.2 %)
Pleth Variability Index; PVI	103939 (64.9 %)	224594 (63.5 %)	328533 (64.0 %)

The values are the means (SDs), numbers (proportions) or medians [IQRs].

Finger 1 – second finger of the same hand; Finger 2 - third finger of the same hand; Finger 3 - fourth fingers of the same hand.

SpO2 - oxygen saturation; BPM - pulse rate (PR), measured in beats per minute (BPM), which is based on the optical detection of the peripheral flow pulse; PI - perfusion index (PI), the ratio of pulsatile blood flow to nonpulsatile or static blood in peripheral tissue, represents a noninvasive measure of peripheral perfusion; SpHb - total haemoglobin (SpHb), which is measured continuously and noninvasively via pulse CO-Oximetry, typically with a fingertip sensor for adult and paediatric patients; SpOC - oxygen content, which is calculated via the pulse CO-Oximeter using the following equation: SpOC (ml/dL) = 1.31 (ml O2/g Hb) × SpHb (g/dL) × SpO2 + 0.3 ml/dL; PVI - Pleth variability index (PVI), a measure of the dynamic changes in the perfusion index (PI) during the respiratory cycle, which is expressed as a percentage (0–100 %).

### 3.1 The results from correlation and difference analyses (primary endpoint)

**Fig. 4:**
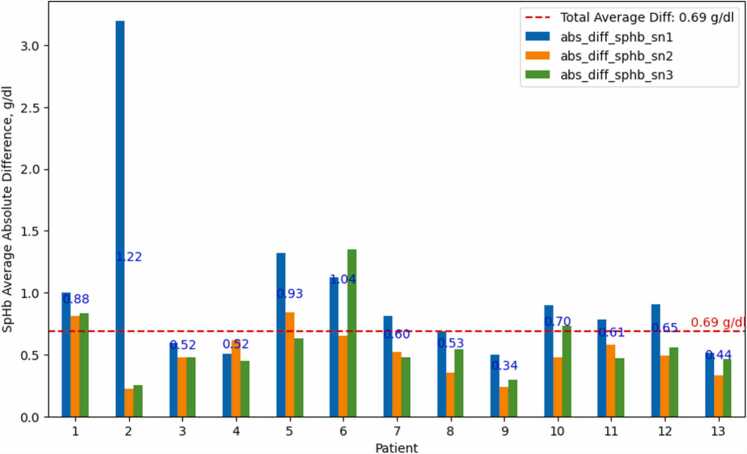
Diagrams show the average absolute differences in SpHb values (_sn1, _sn_2, _sn3) compared with the average of all 3 per patient. The overall absolute average difference was 0.69 g/dl, and the standard deviation was 1.486 g/dl. Based on the combined Kruskal—Wallis H (Kruskal) and Alexander—Govern (ag) tests, the continuous SpHb measurements of the three fingers simultaneously (sphb_sn1, sphb_sn2, sphb_sn3) were significantly different across all patients.

There was a strong positive correlation between the SpHb values obtained from the three fingers simultaneously (*r* = 0.86–0.95). However, on the basis of the results of the combination of the Kruskal—Wallis H (Kruskal) and Alexander—Govern (ag) tests (Fig. 4), the null hypothesis was rejected (*p* < 0.05), which indicates that SpHb from individual fingers of the same hand (sphb_sn1, sphb_sn2, sphb_sn3) significantly differed across all patients: the overall absolute difference between an individual finger’s SpHb and the AvgSpHb of three fingers of the same hand at the same time point was 0.69 g/dl (SD 1.49 g/dl). Statistical analysis was applied for 253 (233, 266) time points (measurements per minute), aggregated from second measurements. Also, see Supplemental Material Appendix B, Fig.B.2 and Table B.1–2.

### 3.2 The results from B&A and Giavarina analyses (secondary endpoint)

Lab-Hb was compared to AvgSpHb at the time points when blood was drawn for Lab-Hb analysis (1-second ABG value), showing a bias of 0.09 g/dL (SD ±0.81 g/dL) and limits of agreement (LOA) from −1.50 to 1.68 g/dL. The repeatability coefficient of AvgSpHb was 3.32, and its precision was 1.18, suggesting relatively consistent differences between Lab-Hb and AvgSpHb, although the LOA indicated worse precision than the manufacturer’s declared specification [15] (Table 2 and Supplemental Appendix B). When comparing Lab-Hb to SpHb from individual fingers, sn1 showed a bias of 1.18 g/dL (SD ±1.07 g/dL) with LOA [−0.91, 3.28], sn2 a bias of 0.07 g/dL (SD ±0.89 g/dL) with LOA [−1.68, 1.81], and sn3 a bias of 0.96 g/dL (SD ±1.76 g/dL) with LOA [−2.50, 4.41] (Table 2 and Supplemental Appendix B). The Giavarina plot comparing Lab-Hb and AvgSpHb differences showed a bias (μ) of 0.57 % (SD 6.97 %) with LOA from −13.09 % to 14.22 %, and corresponding confidence intervals detailed in Supplemental Appendix B. The scatterplot of differences indicated a bias of 0.09 g/dL (SD 0.81 g/dL), with LOA from −1.50 to 1.68 g/dL.

**Table 2:** Bland—Altman statistics.

Bland—Altman Statistics*	SpHb Average	SpHb _sn1	SpHb _sn2	SpHb _sn3
Differences normality test; Shapiro–Wilk statistic (p)’	0.9018 (p = 0.0322 < 0.05); not normal	0.9234 (p = 0.1310 < 0.05); normal	0.8692 (p = 0.0140 < 0.05); not normal	0.9355 (p = 0.1775 < 0.05); not normal
Mean of SpHb	11.1 g/dl	11.2 g/dl	11.4 g/dl	11.2 g/dl
Mean of Lab-Hb	12.0 g/dl	12.4 g/dl	12.1 g/dl	12.1 g/dl
Sample Size (n)	22	19	19	21
DOF	21	18	18	20
Bias; CI in g/dl	0.09 [−0.27, 0.45] g/dl	1.1 [0.67, 1.7] g/dl	0.06 [−0.36, 0.5] g/dl	0.9 [0.15, 1.76] g/dl
Sample SD (s)	0.81	1.06	0.89	1.7
*Lower LOA;* CI in g/dl	−1.5 [−2.12, −0.88] g/dl	−0.9 [−1.8, −0.02] g/dl	−1.6 [−2.43, −0.94] g/dl	−2.5 [−3.89, −1.11] g/dl
*Upper LOA;* CI in g/dl	1.6 [1.06, 2.3] g/dl	3.2 [2.39, 4.17] g/dl	1.8 [1.07, 2.56] g/dl	4.4 [3.02, 5.8] g/dl
Within-Subject SD (Sw)	1.1 g/dl	1.1 g/dl	1.0 g/dl	1.3 g/dl
Repeatability Coefficient (RC)	3.32	3.08	2.93	3.85
Precision	1.17514	1.092155	1.037818	1.364211

* SD – standard deviation; LOA - limits of agreement, DOF - degrees of freedom, CI - confidence intervals; ‘- If differences are not normally distributed, apply logarithmic transformation.

Overall, AvgSpHb outperformed individual finger sensors sn1 and sn3, showing lower bias (0.09 g/dL) and SD (0.81 g/dL). Although SpHb from sn2 had a slightly lower bias (0.07 g/dL, SD ±0.89 g/dL), it exhibited a wider LOA [−1.68, 1.81]. Further details are presented in Table 2, and Supplemental material (Appendix B).

### 3.3 The results from Confidence Intervals (CI) analyses

Our experiment’s Lab-Hb sample size was (n = 22). If we doubled it to (n = 44), the confidence intervals’ widths would decrease from 1.54 standard deviations to 1.05 standard deviations, representing a reduction of 31.4 %. Therefore, maintaining the same measurement conditions while increasing the sample size could improve the confidence intervals by 31.4 %. It means that a larger sample size would not significantly change the final Lab-Hb *vs.* SpHb B&A analysis outcomes [See Supplemental Material Appendix B for more details].

## 4 Discussion

This study investigated the discrepancy between noninvasive SpHb measurements obtained from sensors placed on three fingertips during elective cardiac surgery under CPB. As hypothesized, significant differences were observed between the SpHb values from individual finger sensors and the AvgSpHb across all 253 time points and patients, with an overall absolute difference of 0.69 g/dl (SD 1.49). The most important finding was that AvgSpHb outperformed two of the individual sensors (sn1 and sn3) by demonstrating lower bias (better accuracy) and narrower LOA (greater precision). Although the SpHb measured by sensor sn2 showed a slightly lower bias than AvgSpHb, it had a wider LOA. Overall, AvgSpHb outperformed measurements from all individual sensors. However, these findings should be interpreted with caution, as they lack statistical strength due to the limited number of time points with available Lab-Hb data. Therefore, the main conclusion is that SpHb trends can be significantly affected by the sensor placement site.

Another clinically relevant finding was that the AvgSpHb values demonstrated clinically acceptable accuracy, with a bias of 0.09 g/dL (SD ±0.81 g/dL), given that acceptable accuracy is defined as SD ≤ ±1 g/dL. Since even the most accurate clinical analysers used as reference methods have inherent bias [16], it is widely agreed that accuracy within 1 g/dL is acceptable, especially for noninvasive haemoglobinometers [17]. However, AvgSpHb showed unacceptable precision, with LOA [−1.5, 1.68], meaning the risk of clinically relevant errors remains.

An accuracy of SD ±0.5 g/dL or lower is clinically preferred when Hb levels are below 9 g/dL, as even an error of 0.5 g/dL can lead to erroneous treatment decisions [18]. For example, depending on the patient’s condition and clinical context, an Hb value around 8 g/dL may be used as a transfusion trigger before anticipated or ongoing blood loss. Thus, an overestimated Hb value could delay a necessary transfusion, while an underestimated value might prompt an unnecessary transfusion.

Our study revealed that there was a good positive correlation between SpHb trends. This is an important finding since the absolute SpHb value is not acceptably accurate for anaemia screening and transfusion decision-making [10], [19], but SpHb trend monitoring is currently recommended [3], [10]. However, significant differences were observed between the SpHb of individual fingers and the AvgSpHb of three fingers at all 253 time points. The SpHb from individual fingers of the same hand was significantly different across all patients: the overall absolute difference between an individual finger’s SpHb and AvgSpHb of three fingers at the same time point was unacceptably high (0.69 g/dl; SD 1.49).

Our findings align with previous studies showing that the photoplethysmography (PPG) measurement site is a key factor due to differences in tissue thickness, pigmentation, blood distribution, and vascular movement at the measurement site [20]. Since 2008, numerous studies have examined Masimo’s pulse CO-oximetry technology for its ability to accurately and precisely estimate Lab-Hb. While many reported reasonable accuracy, precision was often insufficient. Importantly, these studies highlighted that patient-specific factors such as peripheral perfusion, use of vasopressors, peripheral vascular disorders, haemoglobin synthesis disorders, and clinical conditions or interventions like cardiopulmonary bypass (CPB) can impair accuracy [3], [5], [10], [21]. For example, Riess et al. found that SpHb was accurate before CPB but less reliable afterward, likely due to anaemia, peripheral vasoconstriction, hypothermia, and other physiological changes in the immediate post-CPB period [4]. Reiss and others caution against relying on SpHb as the sole basis for transfusion decisions [3], [10]. Consistent with this, our results support the conclusion that SpHb is clinically useful mainly for monitoring trends rather than for anaemia screening or guiding transfusions [22]. Finger sites are easily accessible and generally provide good PPG signals. The manufacturer recommends SpHb sensor placement on fingers or toes. Although some studies have used sensors on the wrist or index finger, none have compared multiple sites simultaneously. To our knowledge, no prior study has tested the Masimo Radical 7 monitor with SpHb sensors placed on several fingers at the same time to compare differences in accuracy and precision.

Masimo Corp. has been continuously developing SpHb sensors and software for Radical 7; therefore, the results obtained using different generations of devices and research conducted during different periods may not be consistent or directly comparable. Accordingly, we discuss our study results in the context of theories and models that explain the main possible sources of inconsistency in current techniques and propose solutions that have the potential to improve the accuracy and precision of noninvasive haemoglobin measurement techniques.

### 4.1 Measured and estimated variables

The pulse CO-oximetry technique, which is based on PPG, involves measuring light attenuation by haemoglobin molecules in capillaries of the skin. Light is shone in tissues, and the detected light intensity forms an electric signal—a PPG. A multiwavelength (MW) PPG exploits the wavelength dependence of light penetration in tissues with the aim of measuring light absorption by specific constituents in blood vessels and tissues, including haemoglobin. The most accessible site is the skin [23]. The fingers, palm, face, and ears produce the largest amplitudes of the pulsatile component of the PPG signal. However, motion artefacts are most prominent in the fingers and wrist [20]. Penetration changes depending on the measurement site, with the breast and abdomen possessing greater depths than the arm and forehead do [20].

Based on MW PPG measurements, the Radical 7 monitor extrapolates *estimated Lab-Hb* referred to as total haemoglobin or SpHb. Thus, although SpHb is referred to as *measured*, it is *estimated.* As soon as the Radical 7 entered the market, there was a publication where the authors emphasized that what is actually measured is capillary haemoglobin [24].

These aspects lead to questions as to whether the MW PPG technique is accurate enough for measuring light attenuation by haemoglobin molecules in living tissue and whether methods for the extrapolation of *estimated Lab-Hb* from the PPG signal are correct since both factors may be responsible for the unacceptable reliability of SpHb estimates. There is always space for improvement [1], but state-of-the-art MW PPG techniques are very sophisticated and trustworthy [10], [20].

Since the accuracy of SpHb is acceptable according to most reports and only precision remains an unsolved issue, most of the challenges that are considered reasons for failure to achieve the required accuracy have been overcome [1]. Thus, we believe that a very important “troublemaker” involves the anatomy and physiology of the microcirculation. Technological and methodological solutions require in-depth analysis and modelling of the processes therein. We discuss two aspects. The variability of the capillary network is one aspect, and evidence of its possible impact on SpHb was provided in the present study. The unpredictable transcapillary fluid shifts that might cause “spikes” in local capillary haemodilution have to be identified and adjusted for so that their impact on PPG signal can be filtered.

### 4.2 Variability of the capillary network

The pulse CO-oximetry technique estimates the net volume of haemoglobin in a segment of the capillary network under the SpHb sensor. The *microvasculature* consists of three types of vessels: arterioles, capillaries (true capillaries and metarterioles), and venules. These micro vessels form a network that regulates local blood perfusion and conducts blood–tissue exchange in true capillaries [25].

**Fig. 5:**
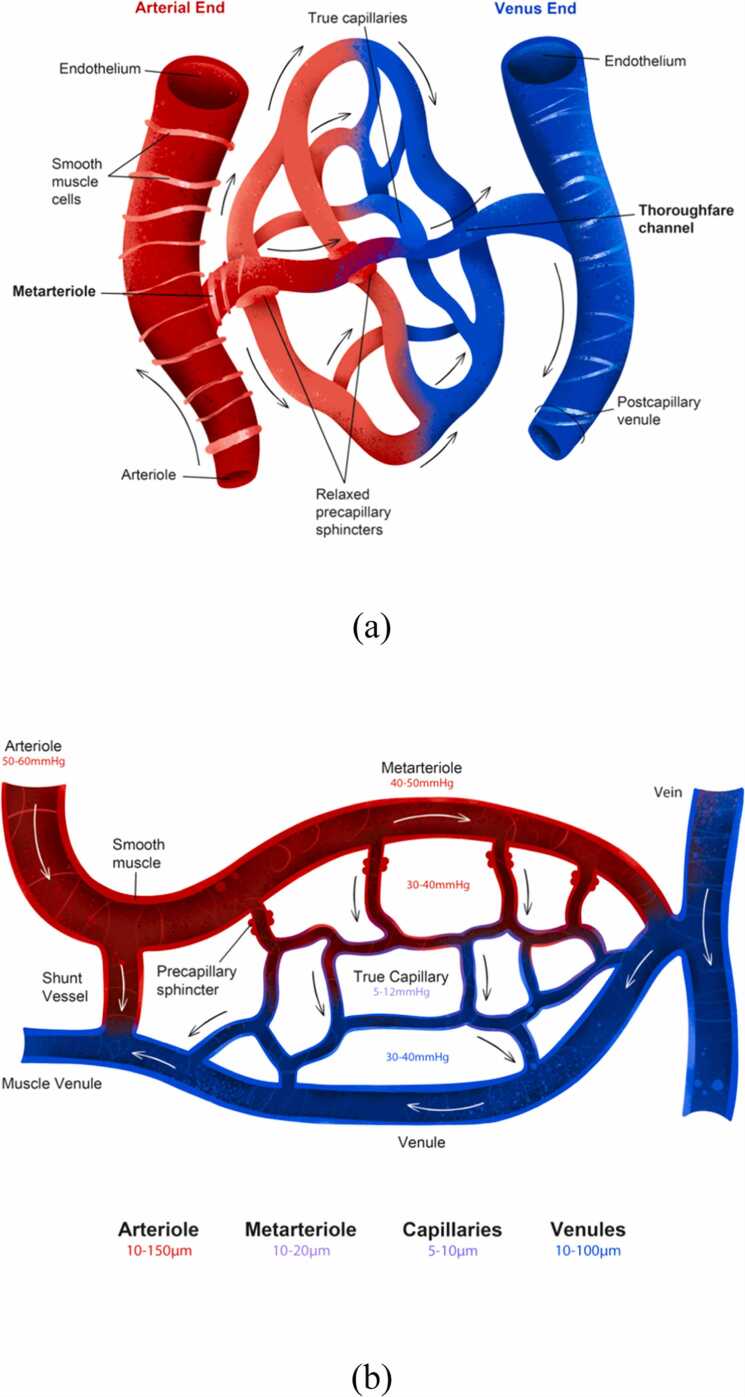
Schematic depiction of well-perfused capillary beds with different configurations of microvessels. (a) Well-perfused true capillaries have fully relaxed sphincters. (b) The pressures within a capillary bed are depicted in a completely different schematic microvascular network than that depicted in Figure (a). Therefore, random sections with different anatomies and everchanging perfusion statuses in the overlapping capillary beds fall into the PPG sensor scan area and cause unpredictable and difficult-to-interpret light absorption patterns and their changes.

**Fig. 6:**
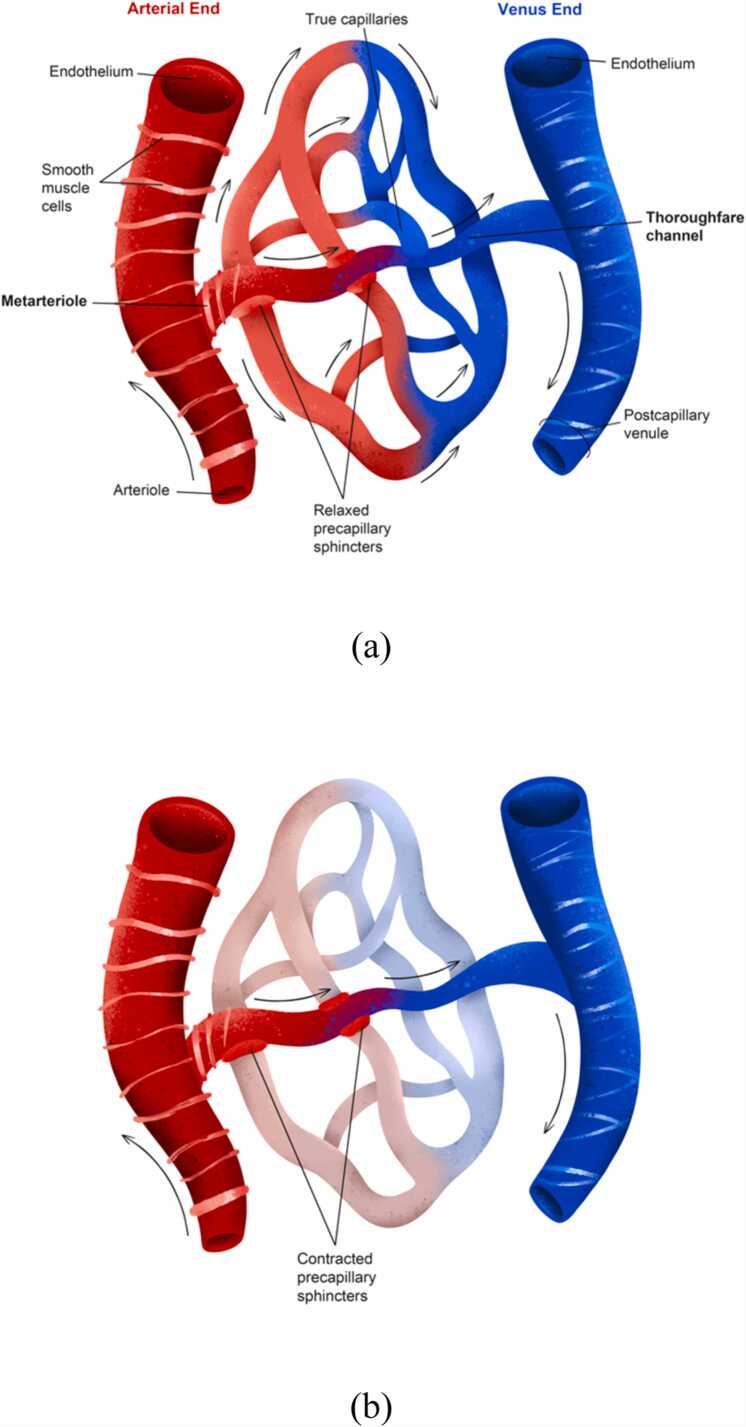
Perfusion of a capillary bed. True capillary perfusion, where transcapillary fluid exchange occurs, depends on the opening of precapillary sphincters. Since no transcapillary shifts occur in the metarteriole (thoroughfare channel or shunt), changes in haematocrit and haemoglobin concentration from the arteriole to the venule depend solely on haemodilution of blood entering from true capillaries. (a) When precapillary sphincters are fully open, true capillaries are perfused. Under normal or dehydrated conditions, more fluid shifts into the tissues than returns to the capillaries, increasing haematocrit and haemoglobin concentrations in true capillaries and metarterioles; thus, the arteriovenous haemodilution difference is negative. Conversely, when tissues are overloaded with fluid, the net fluid shift into true capillaries is greater than into tissues, resulting in greater venular haemodilution and a negative arteriovenous difference. (b) When precapillary sphincters are fully closed, true capillaries are not perfused. The arteriovenous dilution difference is zero because no concentrated capillary blood enters the shunts. However, the PI may remain unchanged or even increase due to increased shunting, potentially leading to misleading clinical interpretation.

Capillary beds have unique configurations (Fig. 5), and they overlap in tissues under the sensor. Thus, the net intracapillary blood volume and haemoglobin mass differ from tissue to tissue. However, perfusion is ever changing even in health, and thus, the net volume of haemoglobin in the same capillary bed also changes (Fig. 6).

The haematocrit (Hct) is the erythrocyte volume fraction of the blood volume. Swan and Nelson suggested in 1971 that capillary beds are the only places in the cardiovascular system where Hct can differ from that in large blood vessels. The capillary Hct is 1/2–1/3 of the large vessel Hct [26]. There are significant differences in Hct between blood vessels of different sizes—the *f-*ratio—due to the Fahraeus effect [27]. The *f*-ratio mean value varies between 0.54 and 1.35 [28]. The continuously changing perfusion of capillary beds results in a changing net radius of perfused capillaries and thus an unstable *f* ratio.

To adjust for the Fahraeus effect, in addition to the in-factory calibration for changes in the perfusion index (PI), an *in vivo* adjustment mode was installed in the Radical-7 monitor in 2012 (*in vivo adjustment* feature in the Masimo radical-7TM. White paper from Masimo Corporation, Irvine, CA) [15]. This implies automatic adjustment of SpHb after the invasive Lab-Hb is entered. An improvement in accuracy was not consistent with conflicting results from other studies [29]. Surprisingly, in some reports, the precision has even worsened in this mode of operation [30]. The need for monitor recalibration when pathophysiological changes occur, e.g., after the induction of anaesthesia, adds to uncertainty [31].

Thus, we suggest that a technique for monitoring changes in the *f*-ratio must be developed since it would allow the adjustment of SpHb estimates accordingly.

### 4.3 Transcapillary fluid shifts

Conceptually, abrupt but short-lasting changes in transcapillary fluid equilibration, e.g., during capillary refill, can significantly change haemodilution in capillaries under the SpHb sensor and produce outliers in B&A plots. Transcapillary fluid equilibration is extremely intense because approximately 95 % of fluid exchange between the blood and tissues takes part in capillaries, while they contain only 3–5 % of the whole-body blood volume.

The findings of our pilot studies support our transcapillary reflux model-based hypothesis that SpHb can be affected by hard-to-predict haemodilution in capillaries due to changes in the transcapillary fluid filtration absorption ratio (FAR) [6], [7], [8]. This may explain why some reports have shown that SpHb is higher than Lab-Hb [32], [33], [34], whereas others have shown the direct opposite [11], [35], [36].

**Fig. 7:**
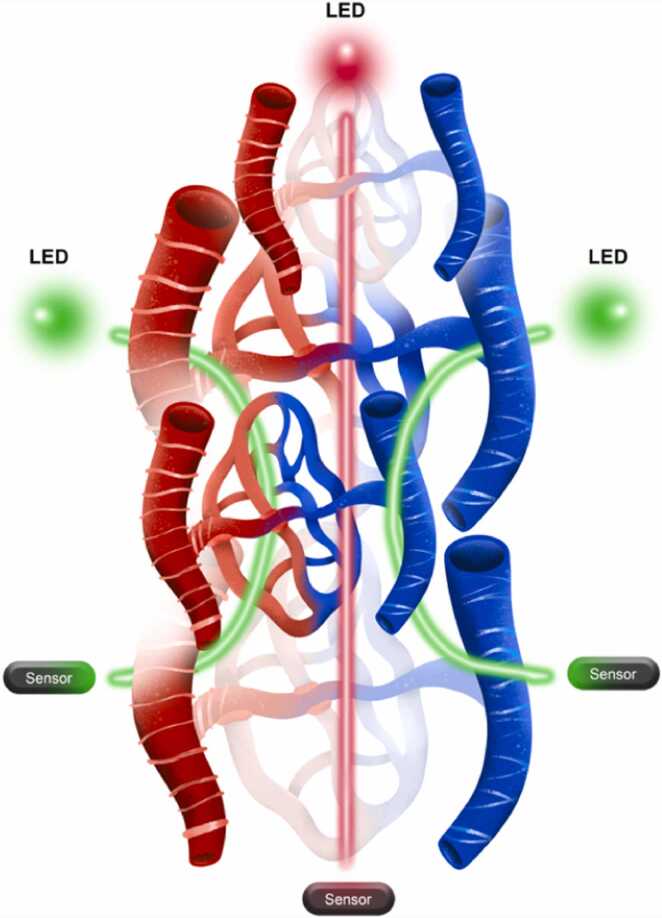
Schematic illustration of a ring-shaped array of sensors that scan different depths and sites of tissues, and the average of their signals is used for extrapolation of noninvasive Hb estimates.

Transcapillary fluid shifts cause the shift in arteriovenous haemodilution from positive during crystalloid infusion to negative after the end of infusion [37]. Moreover, in the steady state, the arteriovenous difference is negligible [34], [38]. The evidence that the perfusion index (PI) can affect the accuracy of SpHb leads to the theory that changes in sympathetic tone that affect the PI [39] can also affect the FAR and thus haemodilution in capillaries, as evidenced by changes in arterio-venous haemodilution [6], [8]. We suggest that better techniques for monitoring transcapillary fluid equilibration and detecting related “spikes” in capillary haemodilution can minimize their effects on SpHb and its trends (Fig. 7).

This study has several strengths, including its within-person design, high volume of data, and use of a state-of-the-art haematology analyser as a reference standard. However, it also has limitations, such as a small sample size and non-standardised blood sampling times. Studies with larger cohorts and a priori sample size calculations, presumably based on our results, are needed to validate the findings more robustly. For now, our results should be interpreted with caution.

## 5 Conclusion

Despite these limitations, our study demonstrates that sensor placement site can impact the accuracy and precision of SpHb in estimating Lab-Hb, with substantial inter-site variability among fingertips. The average SpHb from three adjacent finger tips appears to be more clinically trustable in estimating Lab-Hb compared to individual fingertip. Although averaging SpHb improved the accuracy, the prohibitive cost of multiple disposable SpHb sensors makes this approach clinically impractical. Therefore, we recommend developing reusable MW PPG sensor arrays as a cost-effective solution that could provide spatially averaged SpHb estimates while maintaining economic feasibility for routine clinical use.

## CRediT authorship contribution statement

**Tomas Jovaisa:** Writing – original draft, Validation, Supervision, Methodology, Investigation. **Karolis Urbonas:** Writing – review & editing, Software, Methodology, Investigation, Data curation. **Axel Kerroum:** Writing – review & editing, Investigation, Formal analysis, Data curation. **Puodziukaite Lina:** Writing – review & editing, Project administration, Investigation, Data curation. **Nadezda Scupakova:** Validation, Project administration, Investigation, Data curation. **Edgaras Stankevicius:** Writing – review & editing, Supervision, Formal analysis, Data curation, Conceptualization. **Vaidotas Marozas:** Writing – original draft, Validation, Supervision, Software, Formal analysis, Conceptualization. **Gintaras Kalinauskas:** Writing – review & editing, Supervision, Project administration, Investigation, Data curation. **Povilas Andrijauskas:** Writing – original draft, Supervision, Project administration, Methodology, Investigation, Data curation, Conceptualization. **Mindaugas Budra:** Writing – review & editing, Project administration, Investigation, Data curation. **Audrius Andrijauskas:** Writing – original draft, Project administration, Methodology, Investigation, Conceptualization. **Edvin Vasilevski:** Writing – review & editing, Data curation. **Ieva Jovaisiene:** Writing – review & editing, Validation, Project administration, Data curation. **Darius Cincikas:** Writing – review & editing, Investigation, Formal analysis, Data curation, Conceptualization. **Darius Dilijonas:** Writing – original draft, Software, Investigation, Formal analysis, Data curation. **Valaika Arunas:** Writing – review & editing, Project administration, Investigation, Data curation, Conceptualization.

## Statements & declarations

The authors declare that no funds, grants, or other support was received during the preparation of this manuscript.

## Competing interests

The authors have no relevant financial or non-financial interests to disclose.
