# Supplementary material for: The impact of sensor application site on continuous noninvasive haemoglobin trends
Source: Comput Struct Biotechnol J. 2025 Jul 12;28:249–58. doi: 10.1016/j.csbj.2025.07.013 (PMC12284539; doi:10.1016/j.csbj.2025.07.013)
Supplement: Supplementary file 1 — Supplementary material [file mmc1.docx]

**Supplemental material (Appendix A)**

*Clinical management protocol*

*Pre-CPB phase*

A peripheral intravenous catheter was placed, and gravity-driven intravenous (IV) fluid infusion of Ringer acetate malate (Sterofundin^TM^) commenced at a minimal rate (“keep the vein open”). The choice of infusion fluid and its administration intensity throughout the entire study was the choice of the attending anaesthesiologist who did not participate in the study.

To reduce bleeding, 20 mg/kg tranexamic acid was infused. An infusion of 2 g of magnesium sulfate was given as a part of the enhanced recovery after surgery (ERAS) program. Baseline values of SpHb and other available parameters were recorded.

After premedication with 2–5 mg of i/v midazolam and 50–100 mkg of i/v propofol, the radial artery was catheterized in the same arm, which was used for SpHb measurements, and continuous arterial blood pressure monitoring commenced.

After preoxygenation, anaesthesia induction was performed with propofol, fentanyl and rocuronium, and the trachea was incubated. Mechanical lung ventilation in volume control mode with FiO2 ≥ 40% and sevoflurane commenced. For anaesthesia maintenance, propofol (20–40 mkg/kg/min) and fentanyl (3–5 mkg/kg/h) were used.

A triple lumen central vein catheter was inserted into the right internal jugular vein, and an additional infusion line was connected. Continuous monitoring of central vein pressure (CVP) was started. The CABG surgery commenced with sternotomy. After surgical removal of the internal mammary artery and full heparinization (350 UI/kg, ACT control), the CPB cannulas were placed, and CPB commenced.

*On-CPB phase*

The CPB system was prefilled with 1250 ml of Ringer acetate and 250 ml of 15% mannitol solution. During CPB, blood flow, oxygenation and carbon dioxide removal were maintained with mild hypothermia (≥33 °C). Propofol (40–60 mkg/kg/min) and fentanyl (3–5 mkg/kg/h) were used for anaesthesia maintenance.

Norepinephrine was infused through the CPB if necessary to maintain the necessary blood pressure. Before the aorta was declamped, the patients warmed, and parallel CPB was used during the reperfusion period. Dobutamine was used to improve cardiac output, and norepinephrine or vasopresin was used to treat vasoplegia. After CPB application was stopped and the heparin effect was fully reversed, the CPB cannulas were removed.

*Post-CPB phase*

Lung ventilation and IV fluid therapy were restarted. An infusion of balanced crystalloid solution Sterofundin was used throughout the study period. Colloids and transfusions of blood products were used on an as needed basis. The end of the study was the time at which the surgical team declared the end of the operation.

**Supplemental material (Appendix B)**

*Data Collection*

A custom desktop software application was developed using Python 3.12.4 to manage and collect SpHb measurements and time points (medical actions) from three Masimo Radical-7 (Radical) monitor devices simultaneously. The Radical devices were integrated using USB and RS-232C data exchange formats.

Data collection occurred between June 27th and August 18th, 2024, resulting in a total of 513,720 measurement points from 12 subjects (4 female and 8 male). Measurements are done per second. The average age of the subjects was 63 years, with the oldest participant being 74 years old and the youngest 49 years old.

Each measurement included the following six ratios:

1. SpO2 - Oxygen Saturation
2. BPM - Pulse Rate (PR), measured in beats per minute (BPM), based on the optical detection of peripheral flow pulse.
3. PI - Perfusion Index (PI), the ratio of pulsatile blood flow to non-pulsatile or static blood in peripheral tissue, representing a noninvasive measure of peripheral perfusion.
4. SpHb - Total Hemoglobin (SpHb), measured continuously and noninvasively using Pulse CO-Oximetry, typically with a fingertip sensor for adult and pediatric patients.
5. SpOC - Oxygen Content, calculated via the Pulse CO-Oximeter using the equation: SpOC (ml/dL) = 1.31 (ml O2/g Hb) x SpHb (g/dL) x SpO2 + 0.3 ml/dL.
6. PVI - Pleth Variability Index (PVI), a measure of the dynamic changes in the perfusion index (PI) during the respiratory cycle, displayed as a percentage (0-100%).

All collected data, encompassing demographic details, SpHb readings from each finger, and ABG hemoglobin levels, were securely stored in an electronic MySQL 11.3 database. Each participant was assigned a unique study identification number to ensure patient confidentiality.

The suffixes _sn1, _sn2, and _sn3 denote the Radical devices used for measurements. These devices were randomized across different fingers for each patient.

Table B. 1 Devices randomization across different fingers per Case

| **Case 1** | **Case 2** | **Case 3** | **Case 4** | **Case 5** | **Case 6** | **Case 7** | **…** | **Case 12** |
| --- | --- | --- | --- | --- | --- | --- | --- | --- |
| Finger2 | Finger3 | Finger1 | Finger2 | Finger3 | Finger1 | Finger2 | … | Finger3 |
| Finger1 | Finger2 | Finger3 | Finger1 | Finger2 | Finger3 | Finger3 | … | Finger1 |
| Finger3 | Finger1 | Finger2 | Finger3 | Finger1 | Finger2 | Finger1 | … | Finger2 |

**Motivation for comparison metrics**

*Hypothesis testing*

The choice of statistical test depends on the specific characteristics of the data and the assumptions we can make^[[1]](#footnote-1)^. Given that we have numerical data and three groups, it is crucial to check for normal distribution. If the data is normally distributed, parametric tests can be applied. If not, non-parametric tests are more appropriate.

Multiple group comparison tests, such as one-way analysis of variance (ANOVA), multivariate analysis of variance (MANOVA), Kruskal-Wallis test, or Alexander-Govern test, can compare more than two numerical data sets.

Statistical hypothesis tests were conducted separately for each patient. We performed Levene’s test for homogeneity of variances and the Shapiro-Wilk test for normality.

The normality test failed for all patients, precluding the use of the ANOVA test. Additionally, multivariate normality and homogeneity of variance-covariance tests failed for most patients.

Given these results, the Kruskal-Wallis H Test or the Alexander-Govern test is the most statistically reliable test. These non-parametric tests are ideal for comparing three or more independent groups (i.e., different devices measuring SpHb) and do not assume normality.

*Bland-Altman Analysis*

The Bland-Altman analysis is a frequently applied technique in studies investigating the agreement between two methods of exact medical measurement. In our study, we measure hemoglobin (Hb) using a traditional Lab test (mix ART and VEN) and the SpHb average, calculated from three different finger measurements, each using a separate Radical device. In the following years, the Bland-Altman method has become the most appropriate way of determining the limits of agreement (LOA) between measurements^[[2]](#footnote-2)^

One of the critical problems in the Bland-Altman analysis is the need to meet the assumption of normal distribution. The continuous measurement variables need not be normally distributed, but their differences should be. We performed a Shapiro-Wilk test for normality, with a statistic of 0.9018 and a p-value of 0.0322. The Test Statistic (0.9018) indicates how closely the data follows a normal distribution. A value closer to 1 suggests a better fit to normality. P-value (0.0322) determines the significance of the test. Typically, a p-value less than 0.05 indicates that the null hypothesis (that the data is normally distributed) can be rejected. We reject the null hypothesis since the p-value is 0.0322, which is less than 0.05. This suggests that the diffs are not normally distributed. The assumption of normal distribution is not met, so we logarithmically transformed the data^[[3]](#footnote-3)^. The sample size is small (n=22), because the sample size is n <30, we are using the ***t*-distribution** to calculate the confidence intervals.

Giavarina analysis is identical to Bland-Altman analysis except that it accounts for heteroscedasticity. It uses percentage differences (relative to the means) on the y-axis instead of raw differences. It was published in Giavarina’s 2015 paper data^[[4]](#footnote-4)^-^[[5]](#footnote-5)^. The sample size is small (n=22), because the sample size is n <30, we are using the ***t*-distribution** to calculate the confidence intervals.

Giavarina analysis is identical to Bland-Altman analysis except that it accounts for heteroscedasticity. It uses percentage differences (relative to the means) on the y-axis instead of raw differences. It was published in Giavarina’s 2015 paper^[[6]](#footnote-6)^.

*Data Cleaning*

The data were cleaned and transformed using Structured Query Language (SQL):

1. Cleaning manually entered Lab test values in time points to ensure unified and agreed data format. It had no impact on data accuracy, only fixed the data structure.
2. Converting date formats to ensure data consistency - '%Y-%m-%d %H:%i:00'
3. Measurement of SpHb was done per second, data was aggregated to minute averages.
4. Data was converted from key value format into flat format, per all measurements.
5. Each measurement where assigned postfixes, describing that device were used for measurement - *sn = '1000269229' or sn = '1000000330' (_sn1); sn = '1000146474' (_sn2); sn = '3000003611' (_sn3)*
6. Simple and absolute differences of each measurement per each devise (_sn1 … 3) from average measurement values calculated. Formulas examples:
   1. *sphb_avg = (sphb_sn1 + sphb_sn2 + sphb_sn3)/3); sphb_sn1_diff_from_avg = sphb_avg - sphb_sn1; sphb_sn1_diff_from_avg_abs = abs(sphb_avg - sphb_sn1)*);
   2. Also, total average differences were calculated: *sphb_sn1_diff_from_avg_abs_avg = (sphb_sn1_diff_from_avg_abs + sphb_sn1_diff_from_avg_abs + sphb_sn1_diff_from_avg_abs) / 3*);
7. Measurements were made in complex operative environment pre-CPB (non-invasive devices were creating nose at transition into on-CPB), on-CPB (non-invasive devices had no Hb measurements), and post-CPB (non-invasive devices were creating nose at transition from on-CPB into post-CPB), we had to synchronize SpHb and Lab-Hb (lvHb), time points filtered where both measurements available. Formulas
   1. *sphb_avg_clean = (IF(sphb_sn1 != 0 AND sphb_sn1 IS NOT NULL, sphb_sn1, 0) +IF(sphb_sn2 != 0 AND sphb_sn2 IS NOT NULL, sphb_sn2, 0) +IF(sphb_sn3 != 0 AND sphb_sn3 IS NOT NULL, sphb_sn3, 0)) /(IF(sphb_sn1 != 0 AND sphb_sn1 IS NOT NULL, 1, 0) + IF(sphb_sn2 != 0 AND sphb_sn2 IS NOT NULL, 1, 0) + IF(sphb_sn3 != 0 AND sphb_sn3 IS NOT NULL, 1, 0)));*
   2. *LabHb_diff_sphb_avg = (LabHb - sphb_avg_clean);*
   3. *LabHb_diff_sphb_sn1 = if((sphb_sn1 is null) or (sphb_sn1 =0), Null, (LabHb - sphb_sn1));*
8. SpHb cannot be measured during cardiopulmonary bypass (CBP) as the measurements will be equal to 0, making comparison impossible. Therefore, we will select only Lab-Hb tests conducted outside of CBP or after CBP for comparison.

*Hypotheses:*

- H0: The three dependent measurements (sphb_sn1, sphb_sn2, sphb_sn3) are not significantly different (i.e., they are equal).
- H1: The three dependent measurements (sphb_sn1, sphb_sn2, sphb_sn3) are significantly different (i.e., they are not equal).

*Statistical analysis:*

The correlation analysis reveals that SpHb measurements from the three devices are not identical for all patients. The correlation coefficients range from 0.86 to 0.95, indicating a high positive correlation, though the measurements are not identical.

The dashboards created for exploratory data analysis revealed significant differences in SpHb measurements across the three devices (_sn1, _sn2, and _sn3).


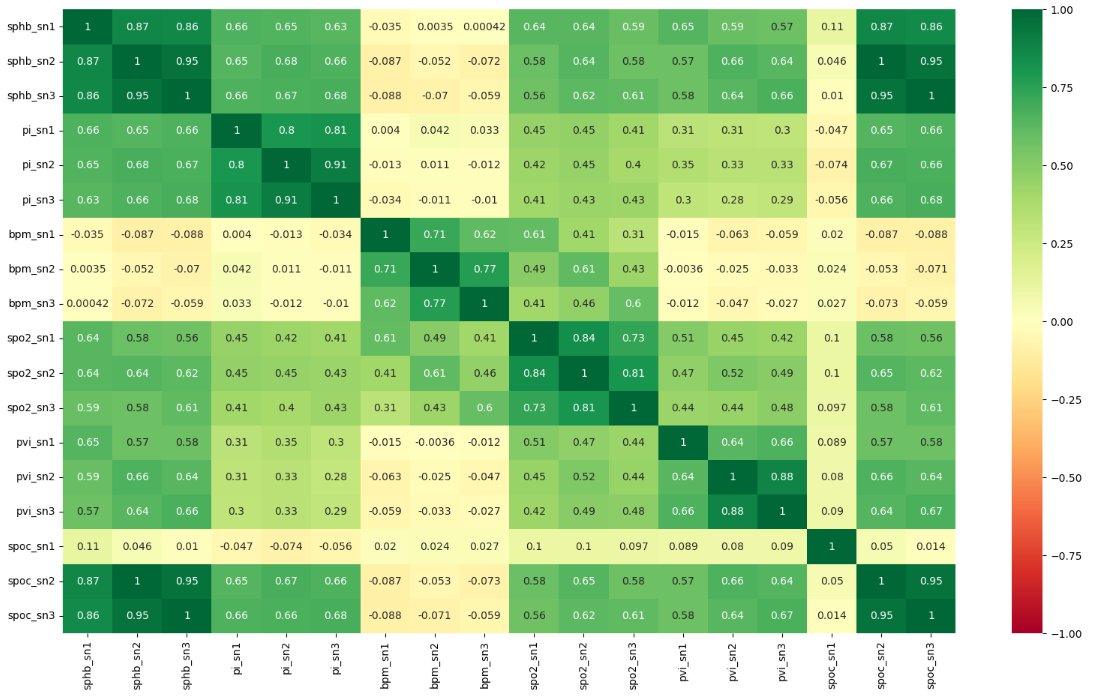


Fig. B. 1 Correlation matrix of all measurements for all patients

The standard deviation of SpHb measurements is also notable. The average SpHb standard deviation is 1.477171 g/dL, with individual device measurements as follows: SpHb_SN1 = 1.642175 g/dL, SpHb_SN2 = 1.715251 g/dL, and SpHb_SN3 = 1.60797 g/dL. The average SpHb value represents the mean of measurements taken from the three devices.


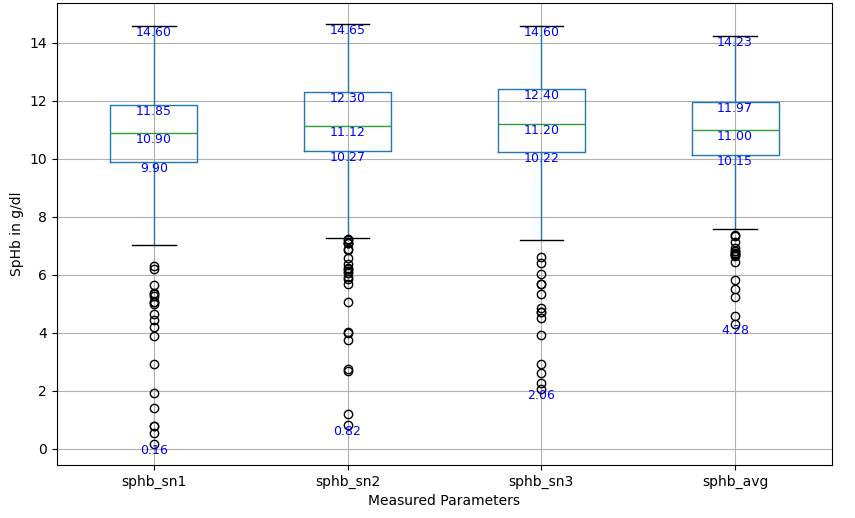


Fig. B. 2 Box-and-Whisker Plot for SpHb_SN1, SpHb_SN2, SpHb_SN3, and SpHb_Avg (All Patients.

The Box-and-Whisker Plot (Fig. B. 3) provides a visual representation of the SpHb data distribution across quartiles: maximum, upper quartile (25%), median (50%), lower quartile (75%), and minimum.

Table B. 2 Box-and-Whisker Statistics

| **sphb** | **min** | **Lower quartile** | **median** | **Upper quartile** | **max** |
| --- | --- | --- | --- | --- | --- |
| sphb_sn1 | 0.160 | 9.900 | 10.900 | 11.852 | 14.600 |
| sphb_sn2 | 0.817 | 10.271 | 11.123 | 12.300 | 14.653 |
| sphb_sn3 | 2.060 | 10.224 | 11.197 | 12.400 | 14.600 |
| sphb_avg | **4**.**285** | **10**.**149** | **11**.**001** | **11**.**972** | **14**.**232** |

The variation in SpHb measurements is significant (Table B. 2), with multiple outliers observed. The lower and upper quartile boundaries for the average SpHb (SpHb_avg) indicate that Radical devices are suitable for trend monitoring. The SpHb values range between 10.149 and 11.972 g/dL, which is medically explainable.

*Hypothesis testing*

The choice of statistical test depends on the specific characteristics of the data and the assumptions we can make [38]. Given that we have numerical data and three groups, it is crucial to check for a normal distribution. If the data are normally distributed, parametric tests can be applied. If not, nonparametric tests are more appropriate.

To compare more than two sets of numerical data, multiple group comparison tests such as one-way analysis of variance (ANOVA), multivariate analysis of variance (MANOVA), the Kruskal‒Wallis test, or the Alexander‒Govern test can be used.

Statistical hypothesis tests were conducted separately for each patient. We performed Levene’s test for homogeneity of variance and the Shapiro‒Wilk test for normality (see Table B. 3).

*Levene's Test for Homogeneity of Variances:*

- *H0: The variances across the groups are significantly different (H0 is rejected).*
- *H1: The variances across the groups are not significantly different (failing to reject H0).*

*Shapiro‒Wilk Test for Normality*

- *H0: The data for sphb_sn1…3 are not normally distributed (reject H0).*
- *H1: The data for sphb_sn1…3 are normally distributed (failing to reject H0).*

Table B. 3 Results of Levene's test for homogeneity of variance and the Shapiro‒Wilk test for normality

| **patient id** | **levene stat** | **levene p value** | **is reject levene test h0** | **shapiro wilk stat sphb sn1** | **shapiro wilk p sphb sn1** | **shapiro wilk stat sphb sn2** | **shapiro wilk p sphb sn2** | **shapiro wilk stat sphb sn3** | **shapiro wilk p sphb sn3** | **is reject sphb sn1 normality test h0** | **is reject sphb sn2 normality test h0** | **is reject sphb sn3 normality test h0** |
| --- | --- | --- | --- | --- | --- | --- | --- | --- | --- | --- | --- | --- |
| 46 | 4.603 | 0.010 | TRUE | 0.714 | 0.000 | 0.709 | 0.000 | 0.641 | 0.000 | TRUE | TRUE | TRUE |
| 48 | 0.253 | 0.777 | FALSE | 0.716 | 0.000 | 0.753 | 0.000 | 0.716 | 0.000 | TRUE | TRUE | TRUE |
| 49 | 0.722 | 0.486 | FALSE | 0.696 | 0.000 | 0.685 | 0.000 | 0.706 | 0.000 | TRUE | TRUE | TRUE |
| 50 | 1.114 | 0.329 | FALSE | 0.750 | 0.000 | 0.723 | 0.000 | 0.737 | 0.000 | TRUE | TRUE | TRUE |
| 51 | 12.755 | 0.000 | TRUE | 0.685 | 0.000 | 0.726 | 0.000 | 0.697 | 0.000 | TRUE | TRUE | TRUE |
| 52 | 1.123 | 0.326 | FALSE | 0.719 | 0.000 | 0.699 | 0.000 | 0.711 | 0.000 | TRUE | TRUE | TRUE |
| 53 | 0.075 | 0.928 | FALSE | 0.720 | 0.000 | 0.694 | 0.000 | 0.675 | 0.000 | TRUE | TRUE | TRUE |
| 54 | 0.291 | 0.747 | FALSE | 0.740 | 0.000 | 0.724 | 0.000 | 0.701 | 0.000 | TRUE | TRUE | TRUE |
| 55 | 1.651 | 0.192 | FALSE | 0.716 | 0.000 | 0.714 | 0.000 | 0.732 | 0.000 | TRUE | TRUE | TRUE |
| 56 | 0.838 | 0.433 | FALSE | 0.673 | 0.000 | 0.699 | 0.000 | 0.701 | 0.000 | TRUE | TRUE | TRUE |
| 57 | 0.872 | 0.418 | FALSE | 0.767 | 0.000 | 0.768 | 0.000 | 0.727 | 0.000 | TRUE | TRUE | TRUE |
| 58 | 0.060 | 0.942 | FALSE | 0.677 | 0.000 | 0.681 | 0.000 | 0.707 | 0.000 | TRUE | TRUE | TRUE |

The normality test failed for all patients, precluding the use of the ANOVA test. Additionally, multivariate normality and homogeneity of variance‒covariance tests failed for most patients:

- Henze–Zirkler multivariate normality test: hz = 452.79, p value = 0.0, normal = false.

Given these results, the most statistically reliable tests are the Kruskal‒Wallis H test or the Alexander‒Govern test. These nonparametric tests are ideal for comparing three or more independent groups (i.e., different devices measuring SpHb) and do not assume normality.

Table B. 4 Kruskal‒Wallis H (Kruskal–Wallis) and Alexander‒Govern (ag) tests

| patient id | Kruskal-Walli’s stat | Kruskal‒Wallis p value | Kruskal‒Wallis interpret | is reject kruskal test h0 | Alexander-Govern stat | Alexander-Govern p value | Alexander-Govern interpret | is reject ag test h0 | Kruskal‒Wallis H Test or Alexander-Govern tests in combination |
| --- | --- | --- | --- | --- | --- | --- | --- | --- | --- |
| 46 | 18.879 | 7.95024E-05 | reject H0 | TRUE | 107.006 | 5.80601E-24 | reject H0 | TRUE | TRUE |
| 48 | 7.682 | 0.021469902 | reject H0 | TRUE | 36.174 | 1.39616E-08 | reject H0 | TRUE | TRUE |
| 49 | 21.936 | 1.72412E-05 | reject H0 | TRUE | 3.206 | 0.201316955 | fail to reject H0 | FALSE | TRUE |
| 50 | 26.484 | 1.77417E-06 | reject H0 | TRUE | 41.612 | 9.20417E-10 | reject H0 | TRUE | TRUE |
| 51 | 36.584 | 1.13713E-08 | reject H0 | TRUE | 47.094 | 5.93699E-11 | reject H0 | TRUE | TRUE |
| 52 | 14.675 | 0.000650598 | reject H0 | TRUE | 21.054 | 2.6805E-05 | reject H0 | TRUE | TRUE |
| 53 | 16.015 | 0.000332924 | reject H0 | TRUE | 1.403 | 0.495795878 | fail to reject H0 | FALSE | TRUE |
| 54 | 5.264 | 0.071922658 | fail to reject H0 | FALSE | 16.642 | 0.000243299 | reject H0 | TRUE | TRUE |
| 55 | 46.124 | 9.64353E-11 | reject H0 | TRUE | 29.888 | 3.23601E-07 | reject H0 | TRUE | TRUE |
| 56 | 29.055 | 4.9056E-07 | reject H0 | TRUE | 24.456 | 4.89153E-06 | reject H0 | TRUE | TRUE |
| 57 | 26.044 | 2.21092E-06 | reject H0 | TRUE | 0.606 | 0.738546358 | fail to reject H0 | FALSE | TRUE |
| 58 | 9.649 | 0.008031797 | reject H0 | TRUE | 32.221 | 1.00741E-07 | reject H0 | TRUE | TRUE |

The percentage of true values in 'reject kruskal test h0' is 91.67%.

The percentage of true values in 'reject ag test h0' is 75.00%.

The percentage of true values in either the 'reject kruskal test h0' or the 'reject ag test h0' columns is 100.00%.

Based on the combination of the Kruskal‒Wallis H (kruskal) and Alexander‒Govern (ag) tests (see Table B. 4), the null hypothesis (H0) is rejected for all patients. This finding indicates that the three measurements (sphb_sn1, sphb_sn2, sphb_sn3) for all patients within the scope of the experiment are significantly different.

*Analysis of differences between Lab-Hb and SpHb (measured using Radical devices)*

SpHb cannot be measured during cardiopulmonary bypass (CBP) as the measurements will be equal to 0, making comparison impossible. Therefore, we will select only Lab-Hb tests conducted outside of CBP or after CBP for comparison.

Table B.5 Lab-Hb and AvgSpHb difference

| **Patient id** | **Lab-Hb (in g/dl)** | **sphb avg clean (in g/dl)** | **Lab-Hb diff sphb avg (in g/dl)** |
| --- | --- | --- | --- |
| 47 | 11.2 | 8.87 | 2.33 |
| 47 | 8.5 | 8.11 | 0.39 |
| 48 | 11 | 11.33 | -0.33 |
| 48 | 9.4 | 9.76 | -0.36 |
| 49 | 11.4 | 10.91 | 0.49 |
| 49 | 14.4 | 10.91 | 3.49 |
| 50 | 13.8 | 13.45 | 0.35 |
| 51 | 12.4 | 10.85 | 1.55 |
| 52 | 14.7 | 13.09 | 1.61 |
| 53 | 13.4 | 12.95 | 0.45 |
| 53 | 10.7 | 11.08 | -0.38 |
| 53 | 11.5 | 11.67 | -0.17 |
| 54 | 11.4 | 10.80 | 0.60 |
| 55 | 12.2 | 11.93 | 0.27 |
| 55 | 9.3 | 13.05 | -3.75 |
| 56 | 12.7 | 10.92 | 1.78 |
| 57 | 14.1 | 12.00 | 2.10 |
| 57 | 14.1 | 12.00 | 2.10 |
| 57 | 10.8 | 9.66 | 1.14 |
| 58 | 13.4 | 11.07 | 2.33 |
| 58 | 13.4 | 11.07 | 2.33 |
| 58 | 11.4 | 10.42 | 0.98 |

Table B. 6 Patient data

| **#** | **id** | **age** | **gender** |
| --- | --- | --- | --- |
| 1 | 47 | 71 | Female |
| 2 | 48 | 58 | Male |
| 3 | 49 | 62 | Male |
| 4 | 50 | 69 | Male |
| 5 | 51 | 0 | Female |
| 6 | 52 | 49 | Male |
| 7 | 53 | 54 | Male |
| 8 | 54 | 74 | Female |
| 9 | 55 | 68 | Male |
| 10 | 56 | 69 | Male |
| 11 | 57 | 59 | Female |
| 12 | 58 | 61 | Male |

Table B. 7 SpHb measurements matching Lab-Hb tests

| **patient id** | **Lab-Hb** | **SpHb Average 3 Devices** | **sphb_sn1** | **sphb_sn2** | **sphb_sn3** |
| --- | --- | --- | --- | --- | --- |
| 47 | 11.2 | 8.87 | NA | 9.20 | 8.54 |
| 47 | 8.5 | 8.11 | NA | 8.41 | 7.80 |
| 48 | 11 | 11.33 | 11.40 | 11.21 | 11.38 |
| 48 | 9.4 | 9.76 | 9.76 | NA | NA |
| 49 | 11.4 | 10.91 | 11.63 | NA | 10.19 |
| 49 | 14.4 | 10.91 | 11.63 | NA | 10.19 |
| 50 | 13.8 | 13.45 | 12.35 | 14.10 | 13.91 |
| 51 | 12.4 | 10.85 | 9.80 | 11.46 | 11.30 |
| 52 | 14.7 | 13.09 | 12.50 | 13.65 | 13.12 |
| 53 | 13.4 | 12.95 | 12.80 | 12.86 | 13.20 |
| 53 | 10.7 | 11.08 | 10.85 | 11.10 | 11.30 |
| 53 | 11.5 | 11.67 | 11.00 | 11.60 | 12.40 |
| 54 | 11.4 | 10.80 | 11.11 | 10.60 | 10.70 |
| 55 | 12.2 | 11.93 | 10.52 | 12.40 | 12.86 |
| 55 | 9.3 | 13.05 | NA | 12.70 | 13.40 |
| 56 | 12.7 | 10.92 | 10.37 | 11.72 | 10.67 |
| 57 | 14.1 | 12.00 | 12.52 | 11.93 | 11.55 |
| 57 | 14.1 | 12.00 | 12.52 | 11.93 | 11.55 |
| 57 | 10.8 | 9.66 | 10.10 | 9.39 | 9.50 |
| 58 | 13.4 | 11.07 | 11.12 | 11.10 | 11.00 |
| 58 | 13.4 | 11.07 | 11.12 | 11.10 | 11.00 |
| 58 | 11.4 | 10.42 | 10.60 | 10.46 | 10.20 |


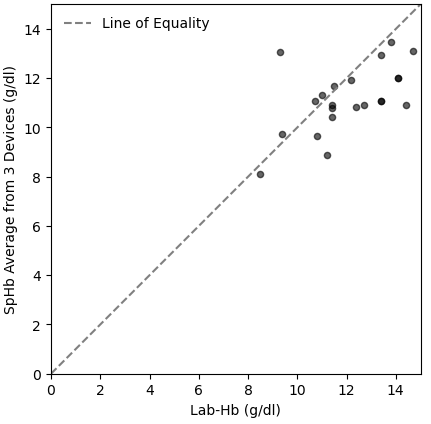


Fig. B. 3 SpHb Average from 3 Devices vs Lab-Hb in (g/dl)

Bland and Altman quantified the difference between measurements using a graphical method. It draws a scatterplot in which the X-axis represented the average [(K1 + K2)/2], and the Y-axis represented the difference (K1 – K2) of two measurements. After the graph is drawn, the mean bias (mean of the K1 – K2) and its confidence limits (limits of agreement) are quantified.

Using Python a one-sample T-test was performed to calculate the mean bias and its SD. To represent mean bias and limits of agreement, we need only the mean of the difference of measurement methods and its standard deviation obtained from one-sample T-test.

Secondly, the data points are restricted using +2 standard deviation (SD) to demonstrate a 95% confidence interval (CI; precisely defined: mean ± 1.96 standard deviations) of distributed data.

One of the critical problems in the Bland-Altman analysis is the need to meet the assumption of normal distribution. The continuous measurement variables need not to be normally distributed, but their differences should.

We performed Shapiro-Wilk test for normality, it’s statistics: 0.9017561648906114, p-value: 0.032235049373301856, see figure (Fig. B. 7)


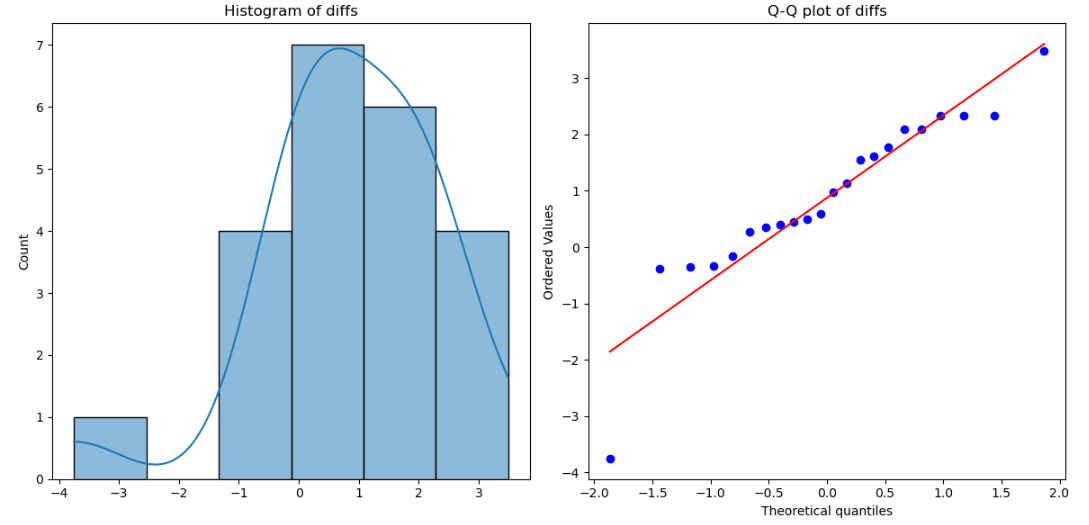


Fig. B.4 Histogram of diffs and Q-Q plot of diffs

Test Statistic (0.9018) indicates how closely data follows a normal distribution. A value closer to 1 suggests a better fit to normality. p-value (0.0322) determines the significance of the test. Typically, a p-value less than 0.05 indicates that the null hypothesis (that the data is normally distributed) can be rejected. Since the p-value is 0.0322, which is less than 0.05, we reject the null hypothesis. This suggests that the diffs are not normally distributed.

The assumption of normal distribution is not met, we transformed logarithmically diffs data.

Here are the measurements taken by the two different Hb measurement methods (Lab and SpHb – using Radical device) plotted against one another (see Fig. B. 8). The sample size is (n=22), because sample size is n=<30, we are using the t-distribution to calculate the confidence intervals.

1.
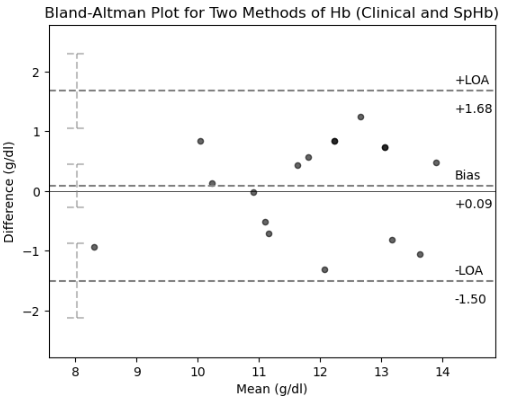

2.
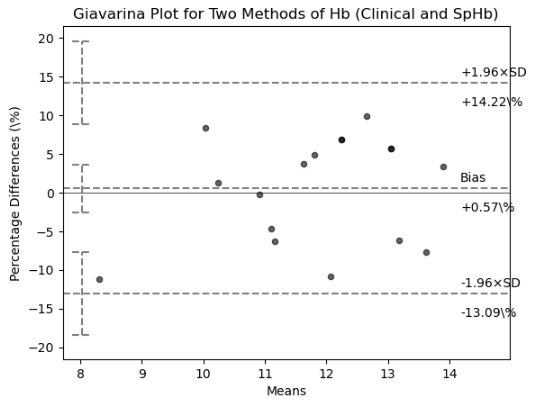


Fig. B. 5 Diagrams showing the accuracy of noninvasive SpHb measurements compared with invasive Lab-Hb measurements: (a) Bland‒Altman plot and (b) Giavarina plot.

The Bland-Altman plot showed the mean bias ±SD between first (Lab) and second (SpHb) Hb levels as 0.0903 ± 0.8116 g/dl, and the limits of agreement were −1.5 and 1.68 (Fig. B. 8).

The scatterplot can be evaluated according to the scatter dispersion. In a good agreement, the scattering of points is diminished, and points lie relatively close to the line which represents mean bias. As a quantifiable measure, mean bias and limits of the agreement give information about the utility of the new measurement method. Regarding our data set, those two methods can be used interchangeably as the limits vary from nearly one g/dl of Hb.

For the differences, bias μ = 0.0903 g/dl and standard deviation std= 0.8116 g/dl

The limits of agreement are LOA upper = 1.6810 g/dl and LOA lower = -1.5004 g/dl; or [-1.5, 1.68]

Giavarina analysis is identical to Bland-Altman analysis except that it accounts for heteroscedasticity. It does this by using percentage differences (relative to the means) on the y-axis instead of raw differences. For the differences, bias μ = 0.5668 % and standard deviation s = 6.9681 %. The limits of agreement are [-13.0907, 14.2243] %; Lower LOA = -13.09, 95% CI [-18.44, -7.74] %; Bias = 0.57, 95% CI [-2.52, 3.66] %; Upper LOA = 14.22, 95% CI [ 8.87, 19.58] %.

*Confidence Intervals (CI)*

The widths of the 95% confidence intervals depend on two factors: the sample standard deviation and the sample size. Obviously, we can’t control the deviation of data but can control the number of samples to test. We can check how the number of samples affect the confidence of experiment results.


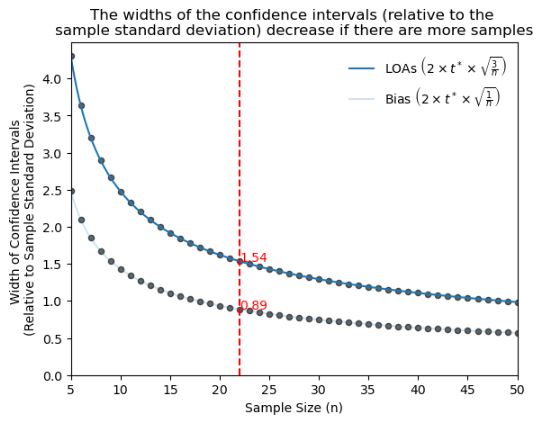


Fig. B.6 Confidence Intervals (CI)

Our experiment’s sample size was (n = 22). If we doubled it to (n = 44), the confidence intervals’ widths would decrease from 1.54 standard deviations to 1.05 standard deviations, representing a reduction of 31.4%. Therefore, maintaining the same measurement conditions while increasing the sample size could improve the confidence intervals by 31.4%. It means that a larger sample size will not significantly change the final analysis outcomes.

Lower LOA = -1.5, 95% CI [-2.12, -0.88]

Bias = 0.09, 95% CI [-0.27, 0.45]

Upper LOA = 1.68, 95% CI [1.06, 2.3]

*Bland-Altman Statistics*

The repeatability coefficient (3.3238) and precision (1.1751) provide insights into the reliability and consistency of the measurements. In the Bland-Altman analysis, the precision value of approximately 1.1751 represents the variability or consistency of the measurements between Lab-Hb and the SpHb average from three finger measurements using Radical devices. A lower precision value indicates higher consistency between the two measurement methods. A precision of 1.1751 suggests that the differences between Lab-Hb and the SpHb average are relatively consistent. The limits of agreement (from -1.50 g/dl to 1.68 g/dl) indicate that most differences between the two methods fall within this range. This level of precision can be considered acceptable or good, depending on the Lab context and the acceptable limits of agreement for the Hb measurements being compared.

Table B. 8 Bland-Altman Statistics

| **Bland-Altman Statistics** | **Value** | **Confidence Intervals (CI) in g/dl** |
| --- | --- | --- |
| Mean of SpHb | 11.1 *g/dl* |  |
| Mean of Lab-Hb | 12.05 *g/dl* |  |
| Sample Size (n) | 22 |  |
| DOF | 21 |  |
| *Bias* | *0.09 g/dl* | *[-0.27, 0.45]* |
| Sample SD (s) | 0.811563632 |  |
| *Lower LOA* | *-1.5 g/dl* | *[-2.12, -0.88]* |
| *Upper LOA* | *1.68 g/dl* | *[1.06, 2.3]* |
| Within-Subject SD (Sw) | 1.19 *g/dl* |  |
| Repeatability Coefficient (RC) | 3.323796502 |  |
| Precision | 1.175139523 |  |

* SD – standard deviation; LOA - limits of agreement, DOF - degrees of freedom*Bland-Altman per Fingers*

Table B. 8 Check for normality of differences using Shapiro–Wilk per Fingers

| **Metric** | **sphb_avg_clean** | **sphb_sn1** | **sphb_sn2** | **sphb_sn3** |
| --- | --- | --- | --- | --- |
| **Shapiro-Wilk Stat** | 0.9018 | 0.9234 | 0.8692 | 0.9355 |
| **Shapiro-Wilk p-value** | 0.0322 | 0.1310 | 0.0140 | 0.1775 |
| **Normality** | Not Normal | Normal | Not Normal | Normal |
| **Bias** | 0.090295 | 1.183947 | 0.066629 | 0.954524 |
| **Sample SD (s)** | 0.811564 | 1.068813 | 0.891605 | 1.764293 |
| **Lower LOA** | -1.500370 | -0.910927 | -1.680917 | -2.503490 |
| **Upper LOA** | 1.680960 | 3.278821 | 1.814175 | 4.412538 |
| **Within-Subject SD (Sw)** | 1.199122 | 1.114444 | 1.058998 | 1.392052 |
| **Repeatability Coefficient (RC)** | 3.323797 | 3.089080 | 2.935394 | 3.858572 |
| **Precision** | 1.175140 | 1.092155 | 1.037818 | 1.364211 |

Method Description

1. Input Data Preparation:
   - The function accepts a DataFrame containing measurements.
   - The columns of the DataFrame represent different tests.
2. Individual Subject Calculations:
   - Means: Calculate the mean of the measurements for each subject.
   - Differences: Calculate the differences between the measurements for each subject.
3. Normality Check:
   - Perform the Shapiro-Wilk test to check for normality of the differences.
   - If the differences are not normally distributed (p < 0.05), apply a logarithmic transformation to the differences.
4. Statistical Calculations:
   - Standard Deviation (s): Calculate the standard deviation of the measurements for each subject.
   - Variance (var): Calculate the variance of the measurements for each subject.
5. Whole Sample Calculations:
   - Mean of Measurements: Calculate the mean of each test across all subjects.
   - Sample Size (n): Determine the number of subjects.
   - Degrees of Freedom (DOF): Calculate the degrees of freedom (n - 1).
   - Bias: Calculate the mean difference between the measurements.
   - Sample Standard Deviation (s): Calculate the standard deviation of the differences.
   - Limits of Agreement (LOA): Calculate the lower and upper limits of agreement (mean difference ± 1.96 * standard deviation).
   - Within-Subject Standard Deviation (Sw): Calculate the within-subject standard deviation.
   - Repeatability Coefficient (RC): Calculate the repeatability coefficient (√2 * 1.96 * Sw).
   - Precision: Calculate the precision (RC / (2 * √2)).
6. Output:
   - The function returns the modified DataFrame with individual subject calculations and a summary DataFrame with whole sample calculations.

Table B. 9 Bland-Altman statistics for different fingers

| **Parameter** | **sphb_sn1** | **sphb_sn2** | **sphb_sn3** |
| --- | --- | --- | --- |
| **Mean (μ)** | 1.1839 g/dl | 0.0666 g/dl | 0.9545 g/dl |
| **Standard Deviation (s)** | 1.0688 g/dl | 0.8916 g/dl | 1.7643 g/dl |
| **Limits of Agreement** | 3.2788 g/dl, -0.9109 g/dl | 1.8142 g/dl, -1.6809 g/dl | 4.4125 g/dl, -2.5035 g/dl |
| **95% of Data Range** | (-0.9109, 3.2788) g/dl | (-1.6809, 1.8142) g/dl | (-2.5035, 4.4125) g/dl |
| **Lower LOA** | -0.91, 95% CI [-1.8, -0.02] | -1.68, 95% CI [-2.43, -0.94] | -2.5, 95% CI [-3.89, -1.11] |
| **Bias** | 1.18, 95% CI [0.67, 1.7] | 0.07, 95% CI [-0.36, 0.5] | 0.95, 95% CI [0.15, 1.76] |
| **Upper LOA** | 3.28, 95% CI [2.39, 4.17] | 1.81, 95% CI [1.07, 2.56] | 4.41, 95% CI [3.02, 5.8] |

Bland-Altman Analysis Method

The Bland-Altman analysis is used to assess the agreement between two different measurement methods. This method involves calculating the mean and difference of the measurements, checking for normality, and determining the limits of agreement.

Method Description

1. Data Preparation:
   - Means and Differences: Calculate the mean and difference of the two measurements for each subject.
2. Normality Check:
   - Perform the Shapiro-Wilk test to check for normality of the differences.
   - If the differences are not normally distributed (p < 0.05), apply a logarithmic transformation to the differences.
3. Statistical Calculations:
   - Bias: Calculate the average difference (bias) between the two measurements.
   - Sample Standard Deviation (s): Calculate the standard deviation of the differences.
   - Limits of Agreement (LOAs): Calculate the upper and lower limits of agreement (bias ± 1.96 * standard deviation).
4. Confidence Intervals:
   - Confidence Level (C): Set at 95%.
   - Critical z-score (z_star): Calculate using the percent-point function of the normal distribution.
   - Confidence Intervals for LOAs: Calculate the confidence intervals for the bias and the limits of agreement using the t-distribution.
5. Bland-Altman Plot:
   - Scatter Plot: Plot the means against the differences.
   - Zero Line: Plot a horizontal line at y = 0.
   - Bias and LOAs: Plot horizontal lines for the bias and the limits of agreement.
   - Confidence Intervals: Plot the confidence intervals for the bias and the limits of agreement.
6. Annotations:
   - Annotate the plot with the bias, upper LOA, and lower LOA values.
7. Output:
   - Display the Bland-Altman plot with the calculated statistics and annotations.
8.
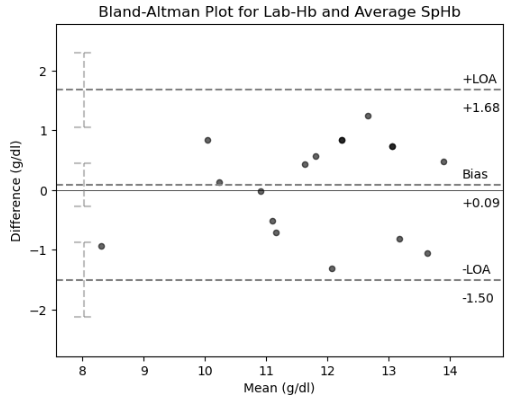

9.
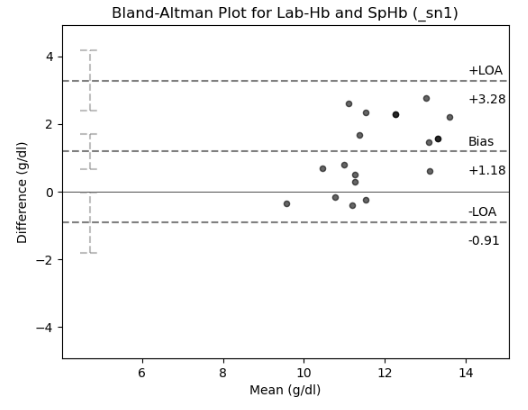

10.
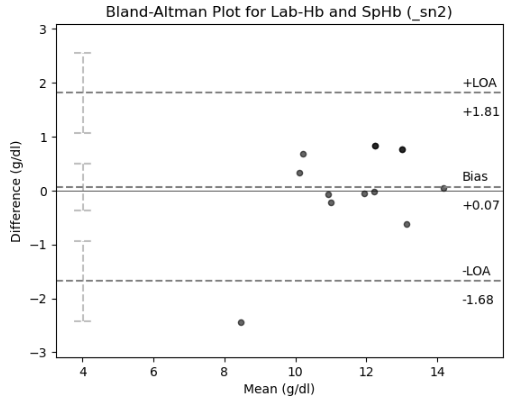

11.
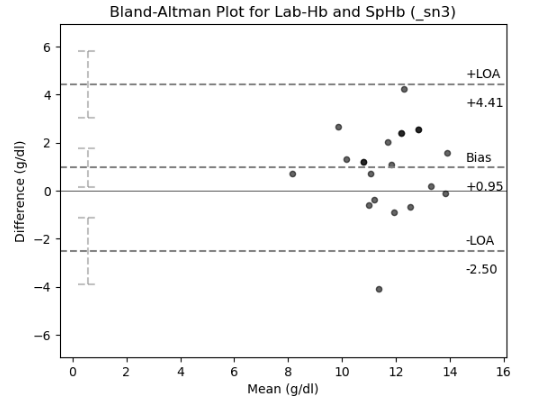


Fig. B.7 Diagrams showing the accuracy of noninvasive SpHb measurements compared with invasive Lab-Hb measurements

Diagrams showing the accuracy of noninvasive SpHb measurements compared with invasive Lab-Hb measurements: (a) Bland‒Altman plot, (b) Bland‒Altman plot (_sn1), (c) Bland‒Altman plot (_sn2) and (d) Bland‒Altman plot (_sn3).

1. Nayak, B. K., & Hazra, A. (2011). How to choose the right statistical test?. Indian journal of ophthalmology, 59(2), 85–86. https://doi.org/10.4103/0301-4738.77005 [↑](#footnote-ref-1)
2. Doğan N. Ö. (2018). Bland-Altman analysis: A paradigm to understand correlation and agreement. Turkish journal of emergency medicine, 18(4), 139–141. https://doi.org/10.1016/j.tjem.2018.09.001 [↑](#footnote-ref-2)
3. Giavarina D. (2015). Understanding Bland Altman analysis. Biochemia medica, 25(2), 141–151. https://doi.org/10.11613/BM.2015.015 [↑](#footnote-ref-3)
4. Giavarina D. Understanding Bland Altman analysis. Biochem Med (Zagreb). 2015;25:141-151 [↑](#footnote-ref-4)
5. Giavarina D. (2015). Understanding Bland Altman analysis. Biochemia medica, 25(2), 141–151. https://doi.org/10.11613/BM.2015.015 [↑](#footnote-ref-5)
6. Giavarina D. Understanding Bland Altman analysis. Biochem Med (Zagreb). 2015;25:141-151 [↑](#footnote-ref-6)
